# Bet-hedging in innate and adaptive immune systems

**DOI:** 10.1093/emph/eoac021

**Published:** 2022-05-24

**Authors:** Ann T Tate, Jeremy Van Cleve

**Affiliations:** Department of Biological Sciences, Vanderbilt University, 465 21st Ave S., Nashville, TN 37232, USA; Vanderbilt Institute for Infection, Immunology, and Inflammation, Nashville, TN, USA; Evolutionary Studies Institute, Vanderbilt University, Nashville, TN, USA; Department of Biology, University of Kentucky, 101 T.H. Morgan Building, Lexington, KY 40506, USA

**Keywords:** immune system evolution, B cells, T cells, macrophages, innate immunity, adaptive immunity, evolutionary medicine, plasticity

## Abstract

Immune system evolution is shaped by the fitness costs and trade-offs associated with mounting an immune response. Costs that arise mainly as a function of the magnitude of investment, including energetic and immunopathological costs, are well-represented in studies of immune system evolution. Less well considered, however, are the costs of immune cell plasticity and specialization. Hosts in nature encounter a large diversity of microbes and parasites that require different and sometimes conflicting immune mechanisms for defense, but it takes precious time to recognize and correctly integrate signals for an effective polarized response. In this perspective, we propose that bet-hedging can be a viable alternative to plasticity in immune cell effector function, discuss conditions under which bet-hedging is likely to be an advantageous strategy for different arms of the immune system, and present cases from both innate and adaptive immune systems that suggest bet-hedging at play.

## INTRODUCTION

Immune systems are in the business of dealing with, and operating within, uncertain environments. Bacterial immune systems have waged endless battles with diverse phages over evolutionary time, while plants and animals face assault from numerous viruses, bacteria, and parasitic eukaryotes. For an individual organism and its offspring, however, the probability of exposure to any one specific parasite is subject to vagaries in environmental conditions, transient epidemiological dynamics, and even random chance.

This uncertainty is reflected in the plasticity of innate and adaptive immune responses. The induction of an immune response relies on the receptor-mediated recognition of non-self or rogue-self antigenic patterns that initiates the production of the appropriate cytokines and effectors. However, a key drawback to plastic inducible responses is that they are reactive rather than preemptive, which can waste precious time against a rapidly proliferating or manipulative pathogen, or create a dangerous temporal lag in response to a rapid subsequent infection by a different pathogen [1]. Plasticity can also be problematic when signals are complex and uncertain, as might be the case in hosts co-infected with worms and germs [2, 3] or when available signals are not specific enough [4, 5].

An evolutionary alternative to plasticity is bet-hedging, where an organism (or immune cell type) might generate diverse offspring phenotypes in anticipation of an uncertain future, so that at least some offspring are well-matched to any future environment [6, 7]. Bet-hedging strategies have long intrigued evolutionary biologists interested in organismal reproduction and phenotype variation and have recently been invoked to explain stochastic phenotype switching in bacteria facing uncertain environments [8, 9]. Under a long evolutionary history of environmental (and microbial) uncertainty, have immune systems evolved to hedge their bets? In this perspective, we first discuss the conditions under which we might expect to see bet-hedging in innate and adaptive immune systems, review evidence for bet-hedging phenomena in macrophages, T cells, and B cells, and outline a way forward for future experimental and theoretical exploration of immune system bet-hedging.

### Bet-hedging

Bet-hedging is the general term for a strategy that maximizes geometric mean fitness across generations by reducing the variance in fitness even though it may reduce the arithmetic mean fitness of an individual or genotype within its lifetime [7, 10–12]. Evolutionarily, bet-hedging is likely to arise when an organism’s environment (and the environment of its offspring) is difficult to predict, or it is infeasible or costly to respond plastically to the uncertainty [13, 14]. Bet-hedging can be conservative, where organisms take on a single phenotype that is slightly but not catastrophically suboptimal in most environments, or it can be diversified where organisms simultaneously invest in a variety of phenotypic strategies that are suboptimal in some environments but optimal in others [6, 10, 15]. Recent theoretical work has emphasized that the evolution of bet-hedging likely depends on the frequency of environmental variability relative to generation time, such that if fluctuations occur too frequently within an organism’s lifetime, the adaptive benefit of bet-hedging dissipates in favor of specialization on one environment [13, 16].

Across ecological systems, diversified bet-hedging has long been recognized as a potential driver of propagule dormancy and seed banking strategies in fungi [17] and plants [15]; for example, the seeds from desert plants may vary in the number of days, months, or years before they germinate (diversified bet-hedging) and in doing so improve the probability that at least some seeds germinate when there is sufficient water available. More recently, the phenomenon of stochastic phenotype switching in bacteria has received attention as a potential example of bet-hedging [18–21], gaining popularity not only for its experimental tractability but also for its role in antibiotic tolerance [22, 23], biofilm persistence [24], and human health. Within an organism, heterogeneity generated by stochastic phenotype switching may also play a role in cancer cell persistence [25, 26]. The role of bet-hedging as an immune system strategy has not been well-explored despite the uncertainty inherent in infection risk, perhaps because the field of immunology has largely focused on the receptors and pathways that give rise to plastic responses. When, exactly, should we expect to see bet-hedging in immune systems, and is there any evidence that immune systems hedge their bets?

### Bet-hedging in immune systems

One of the first studies to highlight the potential for immune system bet-hedging was a theoretical paper explaining the diversity of innate and adaptive immune strategies as a function of cost and parasite frequency and turnover [27]. A form of innate-immune diversified bet-hedging across host generations was predicted to evolve when the pathogen infection is common and pathogen turnover in the environment is relatively slow [27] (Table 1). The intuition in this scenario is that if uninfected periods are long enough to span generations, then it is advantageous to have some offspring who do not pay the cost of innate immunity to specific pathogens even though an infected host may benefit from a rapid immune response.

**Table 1.** Distinguishing the sources and optimization issues of the immunological response to environmental/infection uncertainty

Phenomenon	Strategy	Immunological context	Costs and benefits	Timescale	Notes
Immune phenotype that can shift toward an optimum in response to environment	Reversible plasticity	Immune cell activation; inducible responses rely on recognition and can be turned off or on	Responsive to environmental change if environment is somewhat predictable; can lag behind if environment changes	Within- or trans-generational	The most well-recognized source of response to environmental change (e.g. pathogen exposure)
Immune phenotype is determined by environmental conditions during development of cell or organism	Irreversible plasticity	Immune cell (e.g. helper T cells) polarization and/or differentiation; stable epigenetic state	Beneficial if environment is predictable within a lifetime (cell’s or organism’s)	Within- or trans-generational	Likely costly during co-infection or when developmental signals are heterogeneous
Immune phenotype that appears suboptimal in any environmental condition	Conservative bet-hedging	Specialized response that is not specific to signal despite apparent advantages to specificity	Suboptimal in most environments but minimizes variance in fitness across time	Within- or trans-generational	Unlikely to be favored by selection unless the environment is hopelessly noisy and unpredictable
Proactive variation in offspring immune phenotypes	Bet-hedging (canonical diversified)	Parents anticipate uncertain environments by proactively producing offspring with alternative phenotypes	Beneficial if plasticity is costly or environment changes rapidly	Trans-generational	Each offspring phenotype is better suited to a particular environment but potentially costly in another; ‘bet-hedging’ only if it maximizes E[log(fitness)]
Proactive variation in cell phenotypes	Bet-hedging (diversified)	Bistable generation and persistence of multiple phenotypes regardless of environment; stochastic fate switching. See Table 2 for examples	Beneficial if plasticity is costly or environment changes rapidly	Within-generational or trans-generational (e.g. bacteria)	Bistability generated by ‘adaptive noise’ in gene expression and regulatory machinery

Categories are derived from the evolutionary response outcomes outlined in Botero *et al*. [13]. See also: Mayer *et al*. [27], Viney and Reece [28], Satija and Shalek [29].

The bet-hedging of immunological strategies across generations—for example, variation in how many precious antibodies or antimicrobial peptides a mother deposits into each of her eggs [30]—is not conceptually very different from the better-known examples of intergenerational bet-hedging like propagule dormancy discussed above. However, the potential for bet-hedging to manifest in diverse immune responses *within* an individual host has received far less attention; in other words, when faced with an uncertain infection environment, do hosts hedge their bets by generating multifaceted or diverse cellular-level immune responses over the course of one or multiple infections?

**Figure 1.**
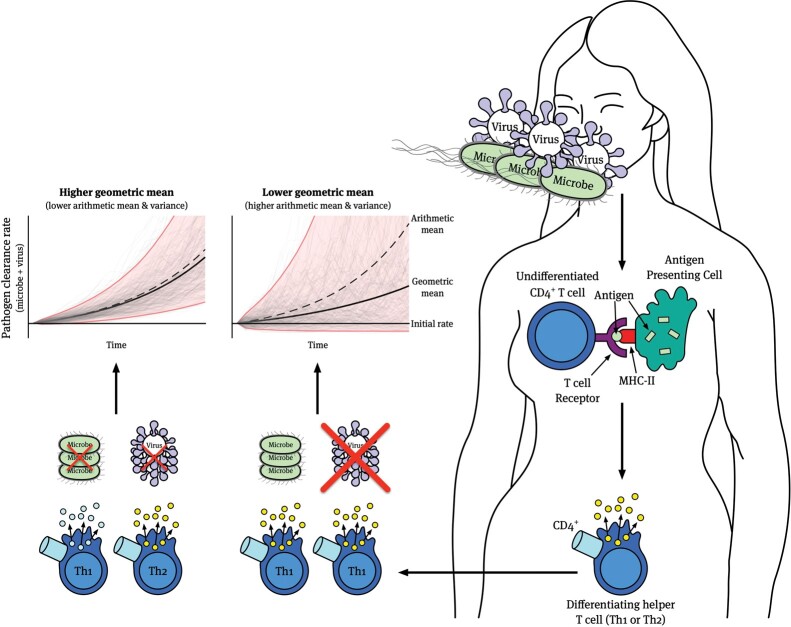
Contrasting the efficacy of immunological bet-hedging (left plot) and polarization (right plot) under uncertain infection conditions. The polarization of immune responses (e.g. by helper T cells) relies on accurate recognition of parasite antigens, which stimulate the production of cytokines that coordinate immune responses to quickly and effectively clear viruses (facilitated by Th1 cells), extracellular microbes and parasites (facilitated by Th2 cells), and other invaders. Polarization and irreversible plasticity of polarized cells may pose an issue, however, if the host is susceptible to infection by multiple types of parasites at once. In cases like these, a polarized response aligned against one parasite type (e.g. Th1 cells against viruses) will result in an initially exponentially growing population of immune cells that effectively clear that parasite type, and hence produce an exponentially increasing clearance rate, but are ineffective at clearing or even impede the clearance of a different type of parasite. This creates substantial variance in pathogen clearance rate where some subpopulations of cells are highly effective, and others are not (right plot). On the other hand, responses that hedge their bets, in terms of producing and maintaining a subpopulation of the ‘wrong’ helper T-cell subtype, may not achieve maximum clearance efficiency against the any single infection but can avoid catastrophically slow responses against a second parasite, reducing overall variance in clearance efficacy. As a result, a bet-hedging strategy (left plot) that has a lower arithmetic mean clearance rate (dashed line) than a polarized response (right plot) can produce a higher geometric mean rate (thick line) due to its lower variance. Assuming clearance rates affect host fitness or cell subtype replication rates within a host, then host genotypes that rely on polarization will have lower geometric mean fitness than those relying on bet-hedging under these conditions. Illustrative simulations were created with a branching process whose growth rate is given by a gamma distribution. The arithmetic mean growth rate and variance in growth rate are lower in the left plot than in the right plot. Gray lines in the plots are sample trajectories and red regions denote 95% intervals.

The advent of single-cell RNA-seq and other fine-scale techniques has revealed substantial variation in phenotypes among immune cells (or even compartments within them) that were previously assumed to belong to homogenous populations (Table 2). Phenotypic variability at the cellular level could be an example of diversified bet-hedging if hosts with this variability more consistently resist infection by unpredictable pathogens at the potential cost of stronger resistance to any specific pathogen variant or type. For example, bet-hedging may be useful in dealing with uncertain infection environments when one strategy may be helpful against a pathogen but actively deleterious against another (see Fig. 1), or when the temporal lag associated with recognition and plasticity gives an intolerable advantage to a pathogen [13]. Just as in the case of stochastic switching in bacteria, we can investigate whether this phenotypic variance or noise [28] increases host fitness. Within a host, for example, immune cell lineages exhibiting more stochasticity might dominate over the course of infection or across host ontogeny while across host generations, selection may favor regulatory elements that promote this cellular bet-hedging. This kind of scenario involves increased phenotypic variance at the cellular level (diversified bet-hedging) but potentially decreased fitness variance among hosts as they more consistently resist pathogen infection.

**Table 2.** Specific examples of phenotypic variance and potential bet-hedging in the immune system

Phenomenon	Strategy	Description	Timescale	References	Notes and unknowns
Phagolysosome Acidification	Bet-hedging (diversified)	Multimodal distribution of phagolysosome pH within a macrophage in anticipation of uncertain bacterial pH optima	Standing variation within or among macrophages	Dragotakes *et al*. [31]	What unit of fitness is optimized? Macrophage replication? Host reproduction?
T-cell polarization but incomplete or alternative fates	Bet-hedging (diversified)	Stochastic variability in regulation or cytokine secretion leads to production of a subset of T cells that take on a state in conflict with the dominant polarization signals/fate	Among T cells, proliferating or differentiating T cells	Feinerman *et al*. [32], Lu *et al*. [33]	If a certain proportion of cells take an alternative phenotype, it is diversifying bet-hedging. If incomplete polarization leads to intermediate phenotypes, may be conservative bet-hedging
Alternative splicing in bone marrow dendritic cells (BMDCs)	Bet-hedging (diversified)	BMDCs respond to LPS stimulation with bimodal variation in abundance and splicing of certain immune-related mRNAs. Variation reinforced by IFN feedback circuits	Among BMDCs (sc-RNA-seq)	Shalek *et al*. [34]	Consequences for fitness are unclear
Antibody cross-reactivity	Bet-hedging (conservative)	Generation of cross-reactive antibodies can produce broad but suboptimal protection	Among B cells	Fairlie-Clarke *et al*. [35]	Fairlie-Clarke *et al*. [35] propose that cross-reactivity might be bet-hedging, but not clear if there is an arithmetic vs geometric fitness conflict, or if it is just an opportunity cost
Plant receptor redundancy, diversity	Bet-hedging?	Plants produce a wide diversity of genome-encoded receptors that can accidentally recognize new pathogen factors	Among hosts, trans-generational	Wu *et al*. [36]	How does this differ from TCR/BCR type diversity? Are they costly to arithmetic fitness?
Using IgM antibodies to buy time while other B cells undergo class switching and affinity maturation	None?	Less specific IgM production buys time for affinity maturation of other B cells	Among B cells	Cobey and Hensley [37]	Not a arithmetic vs geometric fitness dilemma unless the less specific B cells then outcompete the more specific ones

If an immune response engages in bet-hedging, then we might expect to observe stochastic phenotype switching from the dominant effector type or other evidence of a maintenance of phenotypic variation at the cellular level that comes at some immediate cost in certain contexts. For example, an immune response where polarized helper T-cell lineages occasionally produce alternative types (e.g. Th2 lineages occasionally producing Th1 cells) might be effective when hosts are infected simultaneously with multiple pathogen types (Fig. 1). By hedging its bets and producing multiple effector types, an immune response may reduce its variance in pathogen clearance rate across all host tissues since there is a greater chance that the effector type that proliferates in any given tissue or region will be effective against the pathogen in that region. Even if the bet-hedging response loses some short-term efficacy since proliferation may be slower on average due to interference from alternative effector types, the long-term persistence of the response in the host may be enhanced since the variance in clearance rate is lower and the geometric mean clearance rate is higher (Fig. 1). Thus, such bet-hedging immune responses might benefit host fitness.

Table 2 provides examples of observations of cells from both innate and adaptive arms of the immune system where variation might be adaptive due to bet hedging. For example, two separate molecules regulate cellular activation thresholds and responsiveness during the early stages of T-cell activation, allowing the generation of preemptive phenotypic variability among clonally expanding T cells [32]. Meanwhile, tiny differences in feedback circuit signals among otherwise homogenous bone marrow-derived dendritic cells can generate stark bimodal differences in the expression and alternative splicing patterns of immune gene transcripts produced in response to lipopolysaccharide exposure [34]. To date, however, only one immunological phenomenon, variation in macrophage phagolysosome pH, has been specifically investigated as an example of bet-hedging. The multimodal distribution of phagolysome acidification in macrophages may allow those cells to destroy microbes that differ widely in their optimal and inhibitory pHs [31], reducing variance in macrophage success over time as they engulf uncertain microbes.

Cellular-level variability and phenotypic noise among immune cells presents an even more provocative possibility for bet-hedging once one considers that immune cells within a host are capable of proliferating exponentially. In particular, the positive feedbacks that are important in immune cell activation and proliferation [32, 38–41] can generate the kind of exponential proliferation that leads to competition, density-dependence, and Darwinian processes [42, 43] among cell populations within a host. Given variation in proliferation and survival rates among immune cell phenotypes, which are often stabilized for many cellular generations by epigenetic mechanisms, immune cell ‘somatic evolution’ [44] might shape the phenotype distribution of immune cells not only during an acute immune response but also at homeostasis.

Somatic evolution is an example of multilevel selection [45, 46] and entails selection on cellular-level traits both at the between-host (or individual) level to increase host fitness and at the within-host level to increase proliferation and survival of cell lineages in host tissues. A crucial feature of host somatic cell evolution (relative to other kinds of multilevel selection) is that somatic cells persist over multiple host generations only insofar as they permit hosts to survive and reproduce via germ line cells that encode for them [47, 48]. Thus, as is the case with the somatic evolution of cancer cells [49], selection that increases immune cell proliferation and survival at the cost of host fitness must be constrained to act within a single host generation. Cancer cell somatic evolution is driven by a multitude of genetic mutations that disrupt the normal epigenetic regulation of cell proliferation, aging, and programmed death [50]; such mutations constitute a serious breakdown of the cooperation inherent in multicellularity (see ref. [51]). In contrast, immune cell somatic evolution (that does not produce cancer cells) is constrained by the fact that the epigenetic factors underlying phenotypic variation among immune cell lineages are heavily influenced and regulated by neighboring cells whose evolutionary interests are predominantly aligned with the host [47, 48] and whose epigenetic responses evolve due to selection at the host level. In other words, host-level selection should resist epigenetic changes that lead to immune cell proliferation and survival at the cost of host fitness.

Even though the scope for within-host selection on immune cell phenotype is much narrower relative to host-level selection, conflict among these levels might result in phenotypes that have significant adaptive function for the host yet display some apparent dysregulation that is hard to attribute to occasional deleterious mutations. If this dysregulation manifests as cellular-level phenotypic variation, then it may be important to think about how somatic evolution of immune cells might lead to diversifying bet-hedging and phenotypic noise where it otherwise might not benefit the host. A better understanding of bet-hedging dynamics within hosts and across host generations would provide an interesting alternative perspective of the maintenance of immunological variation and seemingly suboptimal immune strategies in natural populations.

### The limits of immunological plasticity and specialization in innate and adaptive immune systems

The vertebrate immune system relies on cell populations from both innate and adaptive arms of the immune system. These cell types, including macrophages, B cells, and T cells, are capable of rapid proliferation after receiving activation signals, but differ in the competitive processes that govern their coexistence with, or dominance over, other clones of their particular subtype. These cell types also differ in the reversibility of their plastic responses and the precious time it takes to achieve a fully activated and/or differentiated state, leading to potentially different fitness costs of plasticity relative to other strategies like diversified bet-hedging or specialization.

Macrophages, for example, can adopt inflammatory or tolerogenic states that are governed by short-term signals (e.g. cytokines) but potentially maintained long-term, and even into subsequent proliferative generations, by epigenetic modifications [52]. Given that a tolerogenic macrophage might protect against lethal sepsis but prove a liability against fungal infection [52], further experimental investigation into the costs and constraints of phenotypic plasticity in macrophages under environmental (i.e. microbial) fluctuations would provide insight into the relative merits of bet-hedging in this form of innate immunity.

In another example, αβT cells, which are reinforced in the thymus by negative selection against self-recognition and positive selection for MHC binding, possess a high degree of specificity for particular antigen-MHC combinations on antigen-presenting cells. A hallmark of helper T-cell biology is their commitment upon activation and the start of proliferation to a polarized state, which is mediated by transcription factors that mutually negatively inhibit each other and the polarized states that they regulate [53]. Polarized cells that are highly activated in one state (e.g. Th1) will proliferate rapidly and outcompete cells from other subtypes (e.g. Th2) that are not as strongly activated [54]. When cytokine signals are clear (e.g. IFNs or IL-12 in response to a viral infection), then polarization of the T-cell population can happen rapidly. If, however, cytokine signals are conflicting or muddled, or if the T-cell population is already strongly polarized, then this process can be less efficient or even lead to incorrect polarization and severe clinical disease, as seen with Hansen’s disease [55] and even some severe COVID-19 cases [56]. This is also a problem with multiple infections, where helminths, for example, can lead to chronic polarization of cells in the Th2 state, limiting the plasticity of the immune system to respond to infections that would benefit from Th1-mediated responses [3]. Theory predicts that the ‘irreversible plasticity’ of T-cell differentiation may still be optimal when environmental predictability is high, but would lose to diversified bet-hedging in less predictable environments [13]. In this case, we might predict that the degree of reversibility in polarization would vary across species in relation to the diversity of the pathogens that routinely infect them.

All daughter cells of a particular B-cell clone bear the same receptor and the same antigenic specificity. B-cell clones compete with other B cells both directly and indirectly at different stages of their development, effector function, and long-term maintenance [57]. The most well-recognized selection process happens in germinal centers, where B-cell lineages undergo somatic hypermutation to improve their affinity for a given antigen. Selection in the germinal centers is mediated by survival and proliferation signals from follicular Th cells, such that those B cells that bind the antigen with higher affinity are more likely to survive and thrive than other cells [58]. Over time, cells bearing higher affinity receptors will proliferate exponentially more rapidly and competitively exclude those that have received weaker proliferation or survival signals. While this process was traditionally believed to result in the local dominance of a single high-affinity clone [59], more recent work suggests that a diverse array of lower affinity clones arise early and are stably maintained within germinal centers [60–62], suggesting that a level of permissiveness in the selection process could enable bet-hedging.

B cells have another mechanism to diversify their portfolio during infection: as they proliferate in response to a specific antigen, some offspring immediately become plasma cells to produce less-specific but rapidly deployed antibodies, while others migrate to germinal centers to begin the slower but more specific affinity maturation and class-switching process. This diversification strategy is likely distinct from bet-hedging (Table 2) because the early plasma cells reduce the cost of inducible specificity by buying time for the affinity maturation process to succeed, rather than serving as an alternative strategy with fitness costs in certain environments. However, mature B-cell effector function can be limited by levels of circulating antibodies [63]. While this has the benefit of conserving energy and preventing immunopathology from excessive responses, it can come at the cost of suboptimal plasticity to antigenic drift [37]. As a result, conservative bet-hedging may come into play if pre-existing B cells that produce somewhat cross-reactive antibodies against a new infection suppress the induction of a more specific and effective *de novo* B-cell response, as suspected in the phenomenon of immunological imprinting against influenza [37].

### Questions for future research

The fundamental similarities in the proliferative and regulatory dynamics of macrophages, B cells, and T cells that contribute to mismatch between cell phenotype and infection environment raise important questions about the potential costs and benefits of plasticity and bet-hedging in these arms of cellular immunity. Given the different selection and regulatory dynamics of immune system components, under what conditions is it a good idea for the immune system to hedge its bets as opposed to commit to a unimodal or plastic response, be that stabilized around the average response or polarized? What is the scope for immune cell bet-hedging generated by within-host selection and somatic evolution and can this somatic evolution explain dysregulation in phenotypes otherwise adaptive for at the organismal level? These questions, and those listed below, are ripe for experimental and theoretical exploration.

1. Under what conditions does immune cell phenotypic variance within a host provide an adaptive advantage?
2. What are the fitness costs of immunological plasticity, for cell lineages and their hosts? Do these accelerate as plasticity increases?
3. When we observe within-host variation in an experimental setting, how can we determine whether the variance derives from a bet-hedging strategy versus other potential explanations? What are the implications for evolutionary medicine or biomedical application?
4. How do growth and virulence properties of pathogens influence the relative merits of developmental stability (canalization), plasticity, and bet-hedging strategies for immune cells within a host? As pathogen diversity or uncertainty increases, how does the optimal strategy or strategies change?
5. How do the Darwinian forces acting on myeloid and lymphoid cell proliferation and differentiation influence the relative advantages and constraints on the stability of canalization, plasticity, and bet-hedging?
6. Across vertebrate taxa, we see substantial variation in immune strategies from MHC allelic diversity to investment in T cells with innate-like versus diversified receptors. Does host life history drive the relative costs and benefits of plasticity versus bet-hedging in the phenotypic regulation of immune cell phenotypes?

From an experimental perspective, answers to these questions would benefit from increased awareness and quantification of variance in cellular or subcellular immune phenotypes and their immediate contributions to resistance against different types or combinations of infections, as exemplified in the study of macrophage phagolysosome bet-hedging [31]. Invertebrate or fast-maturing vertebrate hosts might provide sufficient tractability to couple meaningful proxies of host fitness with the quantification of standing and inducible variation in cell subtypes using scRNA-seq or flow cytometry on samples collected over time. Finally, hosts that have a small cadre of long-standing enemies that require conflicting immune responses (e.g. African buffalo facing mycobacteria and helminths [3]) may provide a good system to test the limits of immune plasticity and identify phenotype noise and cellular-level variation that could be the product of selection for bet-hedging.

While we have focused our discussion on factors conducive to the evolution of immunological bet-hedging in hosts, it is worth recalling that microbes also have bet-hedging strategies at their disposal [8]. Thus, it would be interesting to explore whether plasticity, bet-hedging and specialization strategies practiced by the host immune system influence the (co)evolution of those deployed by pathogens and parasites. For example, the host could limit a pathogen’s geometric growth rate through immune responses that either decrease the arithmetic mean growth rate or increase the variance in the growth rate of the pathogen. The latter could involve deployment of different immune responses at different time points or tissues, or forcing pathogen subpopulations to invest in defense strategies that trade off with growth rate [64]. If high phenotypic variance among immune cells also decreases host fitness variance by, for example, decreasing the opportunity for novel pathogen phenotypes to completely evade host responses, then a potential coevolutionary feedback could occur where microbial diversified bet-hedging could generate selection for host diversified bet-hedging and so on. If host immunological bet-hedging limits or encourages pathogen bet-hedging, then such host responses could be amplified or reversed in a therapeutic setting to limit pathogen related disease. Future empirical and theoretical studies on the coevolutionary implications of bet-hedging would help us better evaluate the feasibility of these outcomes.

## CONCLUSIONS

Although the field of immunology has traditionally focused on the genetics, regulation, and fitness consequences of inducible (plastic) immune responses, evolutionary theory reinforces the idea that bet-hedging can be preferable to plasticity over appreciable swaths of parameter space relevant to defense against parasites and pathogens [13, 27]. Future work on the role of bet-hedging in immune response evolution would benefit from stratifying wild host genotypes or populations by microbe/parasite diversity, predictability, and turnover to test the hypothesis that hosts evolving under less predictable and higher turnover conditions would be under stronger selection for immunological bet-hedging. Detecting bet-hedging in the immune system will likely require assays capable of quantifying phenotypic heterogeneity among individual cells, assessing the relative plasticity of those cells to stimulation, and devising informative measures of arithmetic and geometric fitness at both cellular and host levels. New theory, informed by the biological details of immunological regulation and the relative costs of plasticity, would help us narrow our search for bet-hedging within the overwhelming complexity of the immune system and explain puzzling variation in the dynamics of inducible immune responses. A better picture of the limits of plasticity and bet-hedging in immune systems would inform our understanding of immune system evolution and potentially inspire creative new therapies to improve human health.
